# Study on strength criterion of progressive failure of structural loess under hydraulic action and true triaxial test

**DOI:** 10.1371/journal.pone.0343629

**Published:** 2026-03-16

**Authors:** Juan Fang, Aizhong Luo, Changlu Chen, Weiye Fu, Zijun Zhao

**Affiliations:** 1 College of Civil Engineering and Architecture, Guizhou University of Engineering Science, Bijie, Guizhou, China; 2 Institute of Geotechnical Engineering, Xi ‘an University of Technology, Xi’ an, Shaanxi, China; Henan Polytechnic University, CHINA

## Abstract

The strength behavior of natural loess is governed by its inherent structure and is sensitive to humidity and stress paths, posing challenges for stability assessment in loess engineering. To address this, a three-dimensional strength criterion for structural loess is developed by introducing a shape change coefficient and a structural state parameter, effectively extending classical failure criteria to account for true triaxial stress effects. This criterion is integrated into the Mohr-Coulomb model and implemented in FLAC3D via a custom subroutine for numerical simulation. The model is validated against a series of true triaxial tests under various consolidation pressures (50-300kPa) and intermediate principal stress ratios (b = 0–1). Results show that the proposed criterion accurately captures the stress-strain response and strength evolution of loess, with simulation errors within 5% and strong correlation to experimental data. The study provides a practical theoretical tool for analyzing progressive failure in loess under hydraulic and mechanical actions, with direct relevance to slope, tunnel, and foundation engineering in loess regions.

## 1. Introduction

Loess is widely distributed in the northwest and northwestern regions of China. Due to unique geological sedimentation conditions and climatic environments, loess exhibits distinct characteristics in its microstructure and macro-mechanical properties compared to other soils, making it a typical special soil type. The strength issues of loess are a key focus in the study of rock-soil engineering strength theory, which is crucial for understanding the behavior of rock-soil engineering materials. The strength issues of rock-soil materials have long been a focal point in rock-soil mechanics research. Naturally deposited soils often exhibit primary anisotropy and structural characteristics, as well as secondary anisotropy and structural damage under loading, leading to more complex variations in soil strength patterns. Despite significant progress in research, there are still limitations in both experimental studies and theoretical analyses regarding the strength failure issues of structurally important natural sedimentary loess.

In soil strength theory, Mohr-Coulomb model, a representative of the shear strength theory of civil engineering materials, has been widely applied in rock-soil engineering. However, its application is limited under multi-scale conditions. Many researchers have explored the strength theory of loess under multi-scale conditions, particularly the structural strength theory. Ma Jianquan [**1**] focused on the loess of Heifangtai, Gansu, and studied the characteristics of its strength changes with water content and salt content, considering the effects of salt and water migration. Zhao Yuxin et al. [2,3] investigated the unsaturated strength characteristics of loess in a wide range of saturation and proposed a model for the evolution of unsaturated loess strength indicators over this range. Liu Enlong et al. [**4**] developed a strength criterion for structurally soft soils based on binary medium theory. Matin Liu et al. [**5**] considered the structural influence and established a unified strength criterion for rock-soil materials. Currently, in engineering practice in loess regions, the Mohr-Coulomb strength criterion is still widely used. However, this criterion often deviates π from the strength characteristics of strongly or weakly structured loess under low compressive stress. Revealing the impact of structural properties on loess strength and establishing a reasonable three-dimensional strength criterion is an urgent research topic. Shao Shengjun et al. [**6**] introduced the variation of strength indicators with structural parameters to modify the Mohr-Coulomb strength criterion and applied it to the strength and deformation calculations of structurally soft soils. Due to the unique nature of loess, few destruction criteria have been established, and most are based on experimental results to modify existing strength criteria or empirical simulations. The strength criteria derived from the Mohr-Coulomb law, incorporating structural parameters, have not yet addressed the shape changes of the strength failure surface in the stress space. Jian Yichuan et al. [**7**] proposed a strength criterion for loess based on the true triaxial tests of loess. This criterion is essentially an extension of the Mohr-Coulomb model, with its plane shape satisfying boundary and partial smooth-symmetric conditions. Under triaxial compression, the criterion exhibits smooth-symmetry; under triaxial elongation, it features symmetrical corners and satisfies convexity. Yao Yangping et al. [**8**] developed a generalized strength criterion that combines the generalized Mises criterion and the Matsouka-Nakai criterion, allowing for the selection of appropriate strength criteria for different soils. However, a noticeable research gap persists. Existing criteria often lack a comprehensive integration of the soil’s inherent structural state with the evolution of the three-dimensional failure surface shape under true triaxial stress paths. Specifically, there is a need for a strength criterion that can quantitatively describe how the failure surface on the π plane transitions as the loess structure varies under coupled hydraulic and mechanical actions.

Therefore, this study aims to bridge this gap by establishing a novel three-dimensional strength criterion for structural loess. The primary novelty lies in the explicit introduction of a shape change coefficient and a structural state parameter into the theoretical framework. This approach allows the criterion to capture the evolution of the failure surface geometry linked to structural degradation. The core objectives are: (1) to formulate this three-dimensional structural strength criterion; (2) to implement it within the Mohr-Coulomb constitutive framework for numerical analysis; and (3) to rigorously validate its accuracy by comparing model simulations against a series of controlled true triaxial tests on intact loess.

## 2. Structural strength criteria for loess

### 2.1. Experimental materials, sample preparation, and apparatus

The intact loess tested in this study was obtained from a 6–7 m deep trench in the Qujiang Folk Customs Garden, located in the southern suburbs of Xi’an. The loess is relatively loose, contains a noticeable amount of sand, and exhibits visible calcareous nodules. During sampling and preparation, every effort was made to minimize disturbance. Undisturbed block samples were carefully trimmed and immediately sealed with multiple layers of plastic film and light‑proof tape to preserve the natural moisture and fabric. The loess samples were collected from a non-protected, publicly accessible research area that is dedicated to scientific research activities. According to the management regulations of the test base and relevant local administrative guidelines, sampling for non-commercial, academic research purposes, with the characteristics of small sample volume (total collection volume < 0.5 m³), non-destructive impact on the site (no damage to soil structure or surrounding ecological environment), and compliance with the base’s research access rules—does not require additional permits. The research purpose strictly adheres to academic non-commercial principles, and the sample collection process was supervised and approved by the test base’s management team to ensure compliance with relevant regulations.In the laboratory, cylindrical specimens (70 mm × 70 mm × 140 mm) were prepared for true triaxial testing using manual trimming tools. The basic physical properties of the loess are summarized in Table 1.

**Table 1:** Basic physical properties of the three types of loess.

$\rho_{d}$	/$w_{\text{0}}$%	/$w_{l}$%	/$w_{p}$%	/$I_{p}$%	$e_{\text{0}}$
1.363	15.65	38.1	23.4	14.7	0.986

**Fig 1:**
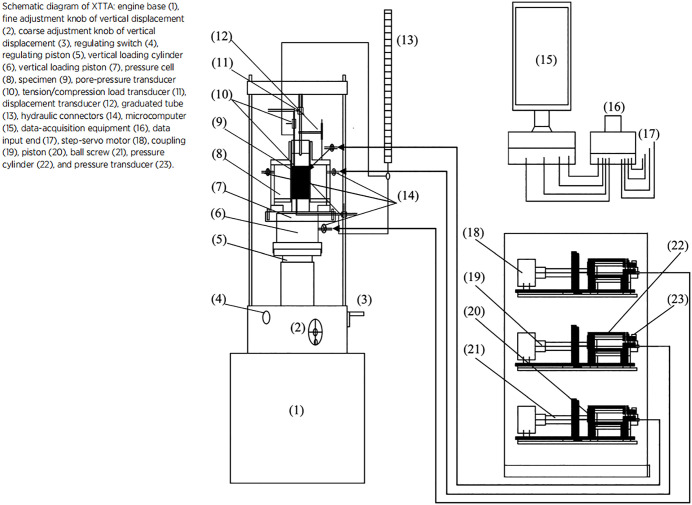
Structure and composition of the true triaxial instrument in Xi ‘an University of Technology [12,13].

**Fig 2:**
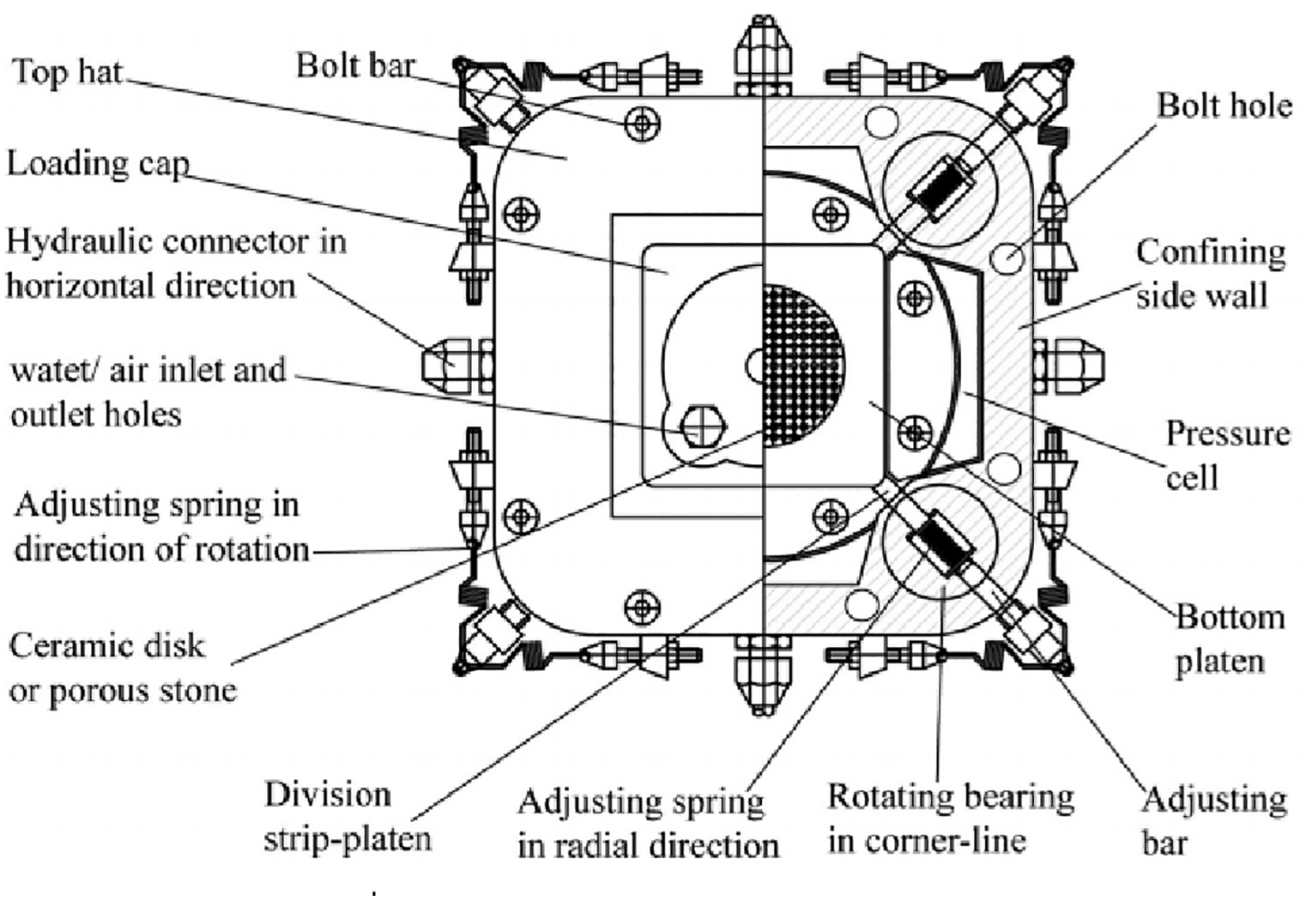
Pressure chamber section [12,13].

The tests were performed using the true triaxial apparatus developed at Xi’an University of Technology (models GT‑1, XGT‑2, and XGT‑3). This apparatus is a composite loading system with one rigid (axial) and two flexible (lateral) loading axes (see Figs 1 and 2). The pressure chamber, originally cubic (70 mm × 70 mm × 70 mm), was later modified to a rectangular shape (70 mm × 70 mm × 140 mm). The specimen is placed in the centre of the chamber; the bottom and top covers are rigid, while the lateral pressures are applied through two pairs of flexible hydraulic bags housed in trapezoidal side chambers. To maintain independent loading on the two lateral faces during specimen deformation, radially extensible and rotationally compliant partitions are installed at the four corners of the pressure chamber. The apparatus comprises three integrated systems: (1)the test host (pressure chamber and loading frames), (2)a servo‑stepper‑motor‑driven hydraulic loading system that independently controls the three principal stresses, and (3)a computer‑based automatic control and data‑acquisition system. The hydraulic loading system uses a servo stepper motor to drive a roller screw, which advances a piston to generate the hydraulic pressure supplied to the axial piston and the lateral flexible bladders. Hydraulic and displacement sensors enable independent measurement and control of all three stress directions throughout the test.

### 2.2. π Strength variation characteristics of loess in the plane

**Fig 3:**
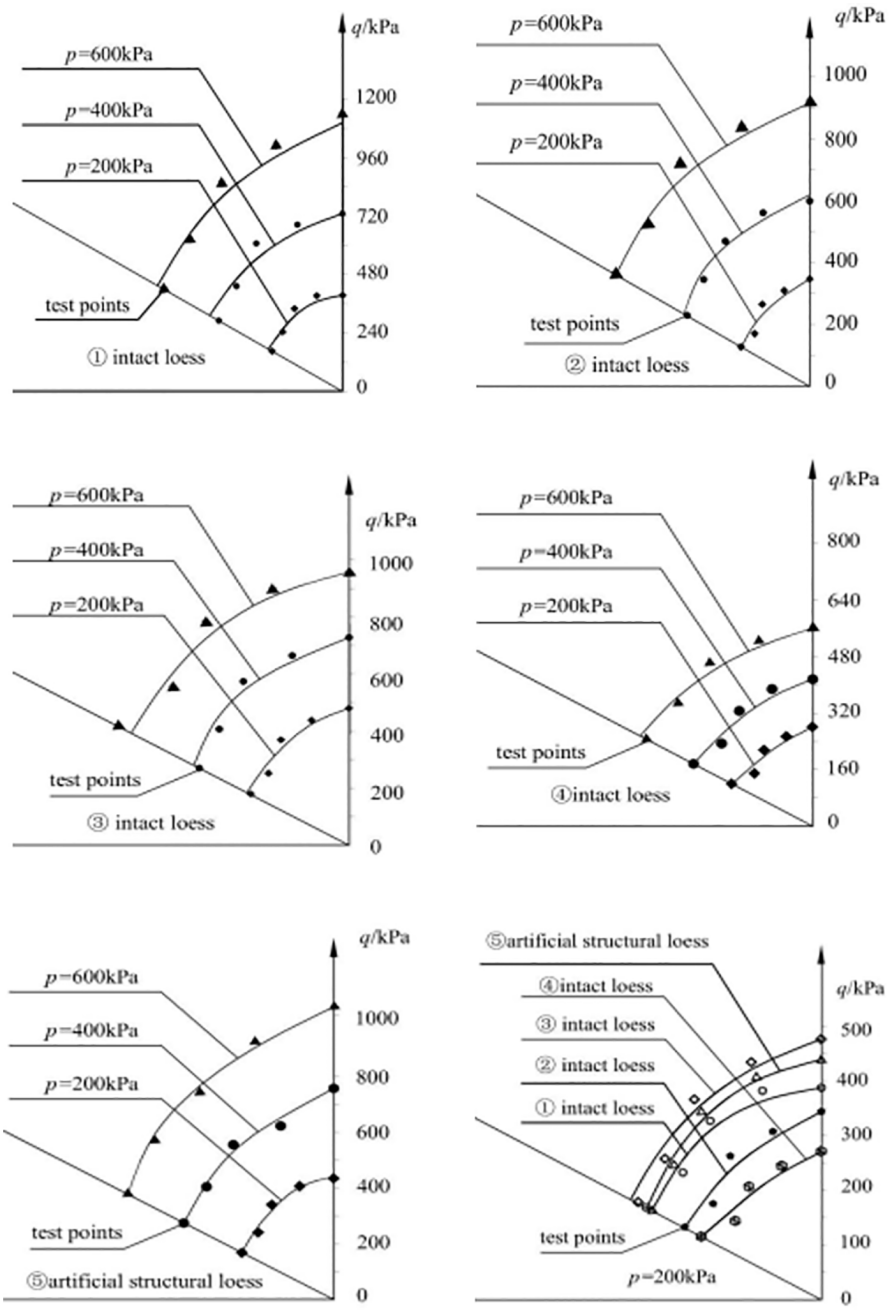
π plane failure lines [11] of several structural loess.

As shown in Fig 3, the strength failure lines of various structural loess on the p-q plane exhibit a good regularity. Although there are some errors in the tests of different loess types, which deviate from the strength failure lines on the π plane, they generally show a good regularity. For loess ① and loess ② with the same structure, loess ① has a lower moisture content and thus greater strength. This indicates that the moisture content significantly affects the strength of undisturbed loess. As the moisture content increases, the strength of undisturbed loess gradually decreases, reflecting the relationship between moisture content and the structural properties of undisturbed loess. The differences also highlight the impact of structural variations.

Under the same ball stress p value (200kPa), the strength failure lines on the π plane of different loess types can be determined. Loess ③ has the highest strength, while Loess ⑤, Loess ①, Loess ②, and Loess ④ have decreasing strengths. For different structural loess types on the π plane, when the moisture content is low (such as in the unconsolidated loess ①), the failure curve on the π plane is nearly circular. As the moisture content increases, the failure curve gradually transforms into a rounded triangular shape (as seen in unconsolidated loess ④). This indicates that the higher the soil’s structural strength, the more circular the failure curve on the π plane; conversely, the lower the structural strength, the closer the failure curve is to a rounded triangular shape. Therefore, the greater the strength of the structural loess, the more the failure line on the π plane approaches a rounded triangular curve. When the structural strength is infinite, the failure line approaches a Mises circle. Conversely, the weaker the structural strength, the more the failure line on the π plane approaches a rounded triangular shape. For different ball stress p values, the failure lines on the π plane of several types of structural loess show that the shear stress increases with the increase in ball stress p. For structural loess with a low moisture content, an increase in ball stress results in a larger increase in shear stress. For structural loess with a high moisture content, an increase in ball stress results in a smaller increase in shear stress. This is primarily due to the varying degrees of structural strength in the loess. When the moisture content is low, an increase in ball stress enhances the hardening of the soil and strengthens the friction between soil particles.

### 2.3. Three-dimensionalization of soil strength criteria

In order to consider the influence of intermediate principal stress in numerical analysis, Gedehus et al. [9] extended the critical state line under conventional triaxial conditions to three-dimensional space and proposed an empirical formula which is expressed as:


$$M\left(\theta_{\sigma }\right)=\frac{2\alpha Mc}{(1+\alpha )-(1-\alpha )sin3\theta_{\sigma }}.$$ (1)

In equation (1) $M_{c}$, represents the slope $\theta_{\sigma }=-\frac{\pi }{\text{6}}$ of the critical state line on the plane α of three-axis compression $\theta_{\sigma }=\frac{\pi }{\text{6}}$; is the ratio of the slope of the critical state line $\theta_{\sigma }=-\frac{\pi }{\text{6}}$ on the plane of three-axis elongation to that on the plane of three-axis compression. According to the Mollhausen criterion, this can be expressed as:


$$\alpha =\frac{M_{e}}{M_{c}}=\frac{3-sin\varphi }{3+sin\varphi }.$$ (2)

In formula (2), $M_{c}$, represents the slope $\theta_{\sigma }=-\frac{\pi }{\text{6}}$ of the critical state line on the plane α of three-axis compression $\theta_{\sigma }=\frac{\pi }{\text{6}}$; $M_{e}$ is the slope of the $\theta_{\sigma }=\frac{\pi }{\text{6}}$ critical state line in the plane of flatness φ in the three-axis elongated state, and is the internal friction angle of soil.

Sheng et al. [9] discussed Equation (2) and believed that Equation α≥0.778 (2$\varphi <22^{o}$) only satisfies the convexity of the yield surface described by Equation (2). Therefore, Sheng proposed a revised formula for Equation (2), which is expressed as:


$$M\left(\theta_{\sigma }\right)=M_{c}[\frac{2\alpha^{\text{4}}}{(1+\alpha^{\text{4}})-(1-\alpha^{\text{4}})sin3\theta_{\sigma }}{]}^{1/4}.$$ (3)

The modified equation (3) α≥0.6 can satisfy $\varphi \leq 48.59^{o}$ the convexity of the yield surface under the condition α=0.6 that (that is), and the proof shows α=1.0that this criterion is the outer line of the Mokken criterion when, and the criterion is the Mises criterion.

### 2.4. Structural strength criteria of loess

**Fig 4:**
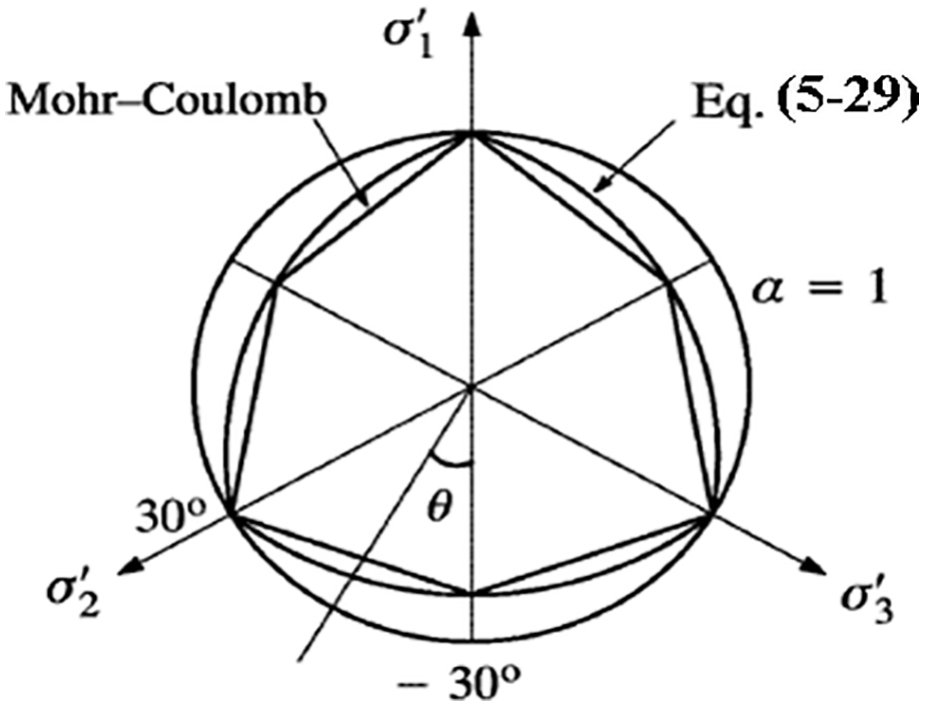
Yield π function on a plane.

**Fig 5:**
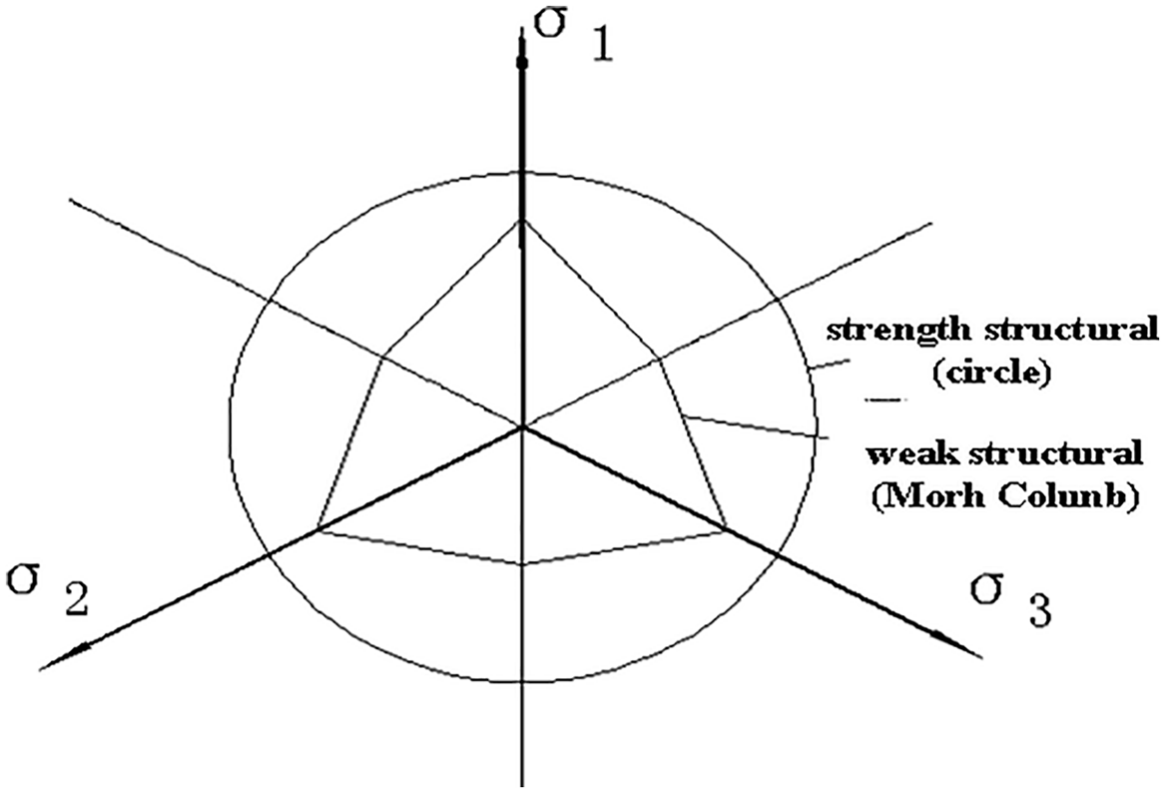
Schematic diagram of the relationship between soil structure and strength failure surface.

Deng Guohua et al. [10] found through true triaxial tests on loess under various moisture content and consolidation pressure conditions that the moisture content and the structural state of the loess are the primary factors influencing the size and shape of the strength failure surface. The increase in moisture content alters the bonding characteristics of π the loess skeleton particles, and the failure of these bonds also leads to changes in the arrangement π of the skeleton particles, ultimately causing damage to the original structure and a reduction in strength. The strength failure surface of soil in the plane transitions from a circular to a Mohr-Coulomb hexagonal shape. Figs 4-5 provide schematic diagrams of the strength of loess with different π structures in the plane. This shows that whether it is changes in moisture content or increased disturbance, the fundamental factor affecting the shape and size of the strength failure surface is the change in π the soil’s structural state. There are two extreme states for this change: when the structure is very weak, the cohesive component contributes little to the soil’s shear strength, while the frictional component contributes significantly, making the strength failure surface in the plane more similar to the Mohr-Coulomb strength surface, meaning the strength during triaxial compression is significantly higher than during triaxial extrusion; when the st*r*ucture is very strong, the cohesive component contributes significantly to the soil’s shear strength, while the frictional component contributes little, making the strength failure surface in the plane more circular, meaning the strength during triaxial compression and triaxial extrusion is essentially the same.

If a structural state variable is defined to characterize the size of π the soil structure, then it satisfies on the plane:


$$r=\frac{q_{s}}{q_{d}}.$$ (4)

**Fig 6:**
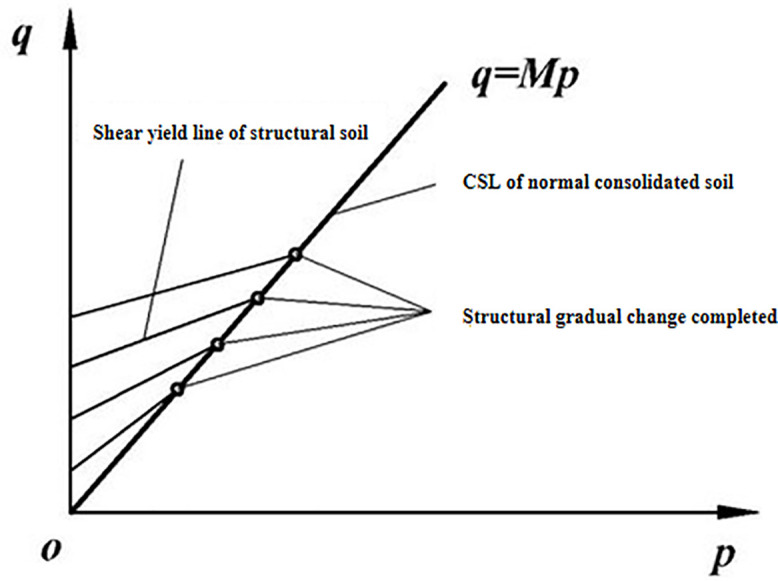
Critical state line of structurally damaged soil.

**Fig 7:**
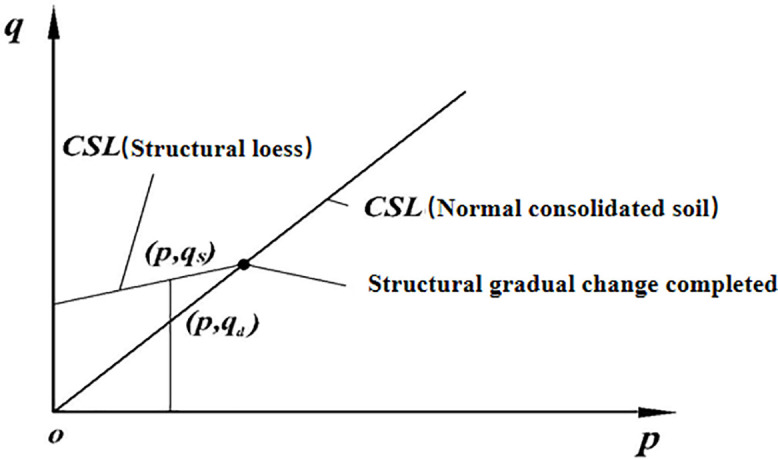
Schematic diagram of critical state between structurally damaged soil and normal consolidated soil.

In equation (4) r, represents the structural $q_{s}$ state variable, represents the $q_{d}$ shear strength of soil with a specific structure, and represents the shear strength of normally consolidated soil under the same ball stress conditions. Based on the variation patterns of the critical state lines of loess in different moisture contents, the critical state line of loess is illustrated in Fig 6. According to Fig 6, the structural state variable satisfies the form shown in Fig 7:

According to Equation (4), the shear strength of any structural soil can be expressed by the shear strength of the structurally completely damaged soil (hereinafter referred to as normal consolidated soil), which is expressed as:


$$q_{s}=rq_{d}.$$ (5)

For normal consolidated soil, under triaxial compressive stress conditions, it satisfies:


$$q_{d}=M_{c}p.$$ (6)

Substituting equation (6) into equation (5), we can get


$$q_{s}=rM_{c}p.$$ (7)

In the general stress state, according to Equation (1), the shear strength of normally consolidated soil is expressed as:


$$q_{d}=M\left(\theta_{\sigma }\right)p=[\frac{2{\alpha_{n}}^{\text{4}}}{(1+{\alpha_{n}}^{\text{4}})-(1-{\alpha_{n}}^{\text{4}})sin3\theta_{\sigma }}{]}^{1/4}M_{c}p.$$ (8)

In the formula $\alpha_{n}=\frac{M_{e}}{M_{c}}=\frac{3-sin\varphi_{n}}{3+sin\varphi_{n}}$,

In the general stress state, the shear strength of structural soil is expressed as


$$q_{s}=[\frac{2{\alpha_{s}}^{\text{4}}}{(1+{\alpha_{s}}^{\text{4}})-(1-{\alpha_{s}}^{\text{4}})sin3\theta_{\sigma }}{]}^{1/4}rM_{c}p.$$ (9)

As can be seen from Fig 6, when the soil is $\alpha_{s}==\frac{3-sin\varphi_{n}}{3+sin\varphi_{n}}$ normal consolidated soil,. When the soil has strong structure, the $\alpha_{s}=1$ failure surface $\alpha_{s}$ is close to the Mises circle of metal. Therefore, the mathematical description of can be constructed as:


$$\alpha_{s}=\frac{r\alpha_{n}}{r\alpha_{n}+1-\alpha_{n}}.$$ (10)

Then equation (10) can be rewritten as:


$$q_{s}=[\frac{2(\frac{r\alpha_{n}}{r\alpha_{n}+1-\alpha_{n}}{)}^{\text{4}}}{(1+(\frac{r\alpha_{n}}{r\alpha_{n}+1-\alpha_{n}}{)}^{\text{4}})-(1-\left(\frac{r\alpha_{n}}{r\alpha_{n}+1-\alpha_{n}}{)}^{\text{4}}\right)sin3\theta_{\sigma }}{]}^{1/4}rM_{c}p.$$ (11)

Equation (11) illustrates the variation pattern of critical state lines for different structural soils under general r stress conditions. To investigate the variation pattern of the structural influence coefficient, is the benchmark α value of normal consolidated soil, the stress $m_{\eta }$ ratio structural parameter r is further incorporated into equation (11). For normally structured soil, where the stress ratio structural parameter is constant at 1, the structural influence coefficient can be expressed as:


$$r=\frac{q_{s}}{q_{d}}=\frac{\frac{\text{1}}{a}m_{\eta }q_{d}-\frac{b}{a}q_{d}}{q_{d}}=\frac{\text{1}}{a}(m_{\eta }-b).$$ (12)

Substituting equation (12) into equation (2) can obtain the variation law of loess critical state line under any structural parameter.

**Fig 8:**
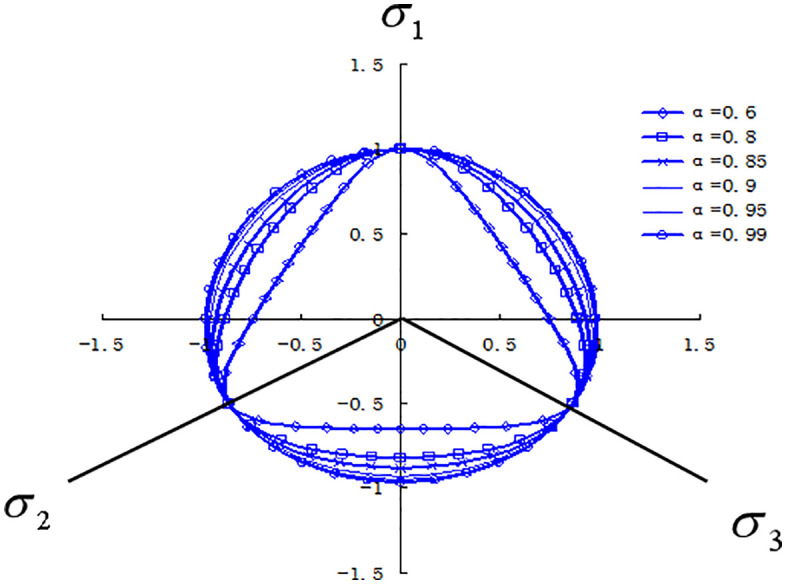
Yielding shapes of different soil planes under the π same structural parameters.

**Fig 9:**
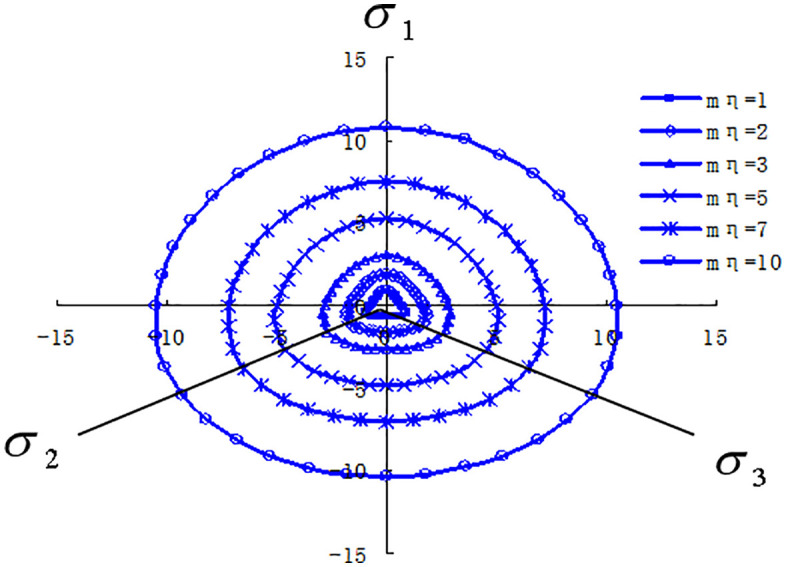
The plane dynamic yield criterion of structural π soil under different structural parameters.

According to equation (12), when the structural influence coefficient is set to 1, the yield π criteria on a plane under different internal friction angle conditions are illustrated in Fig 8. It is evident that when the structural influence coefficient is 1, the strength parameters π reflect the effects of the principal stress in various soil types. By considering the structural influence of soil using equation (12) the strength failure surface on a plane of structurally influenced soil is depicted in Fig 9. As the structural parameter increases, the soil’s strength failure surface transitions from an approximate Mohr-Coulomb criterion unequal-sided hexagon to a Mises circle. As structural damage evolves, the soil’s strength failure surface gradually shifts from a Mises circle back to an approximate Mohr-Coulomb criterion unequal-sided hexagon.

3. True triaxial test simulation of wet load structural constitutive model based on loess

Soil is a three-phase granular material that is distributed in nature over space and time. Different regions and sedimentary periods result in soil with varying mechanical properties. Additionally, different stress conditions of soil can lead to different macroscopic mechanical responses. In past soil mechanics research, the stress conditions of soil were often simplified, and tests were conducted in laboratories using the available experimental instruments, such as direct shear tests and conventional triaxial tests. Although these test conditions correspond to a few natural scenarios, their simplicity has made them widely used in engineering practice. However, as projects have become more complex and structures have grown taller, the stress conditions of soil have become more intricate. To better serve practical engineering needs, instruments for testing complex stress paths have been improved, and advancements in computer technology have made calculations easier. Consequently, researchers have begun to develop instruments for testing under more complex stress conditions to study the deformation and strength characteristics of soil under such conditions, leading to the introduction of true triaxial meters into the research field.

Generally, a true triaxial instrument is designed to independently $\sigma_{\text{1}}$ apply $\sigma_{\text{2}}$ $\sigma_{\text{3}}$ loads in three directions (in the principal stress space), enabling complex stress path tests with different principal stresses. Additionally, the deformations caused by loading in these three principal stress directions must be measured independently. This is a fundamental requirement for a true triaxial instrument. Furthermore, uniform distribution of stress and strain loading, controllable drainage conditions, loading and deformation rates, controllable stress-strain conditions, and a comprehensive control measurement system are all essential criteria for assessing the rationality and completeness of a true triaxial instrument. The process of establishing the soil constitutive model should also reflect the effects of complex stress. To validate the loess structural compression-shear wet structural constitutive model established in this paper, which describes the macro-mechanical behavior of soil under complex stress conditions, a customized constitutive model was numerically implemented using the FLAC3D platform. Based on the strength criteria derived in this paper, which consider the structural characteristics of loess, an external function was written to achieve real-time updates of the model’s calculation parameters. The finite difference method was used to simulate the true triaxial test of collapsible loess, and the results were compared with the data from the true triaxial test.

### 3.1. True triaxial numerical model and loading method

**Fig 10:**
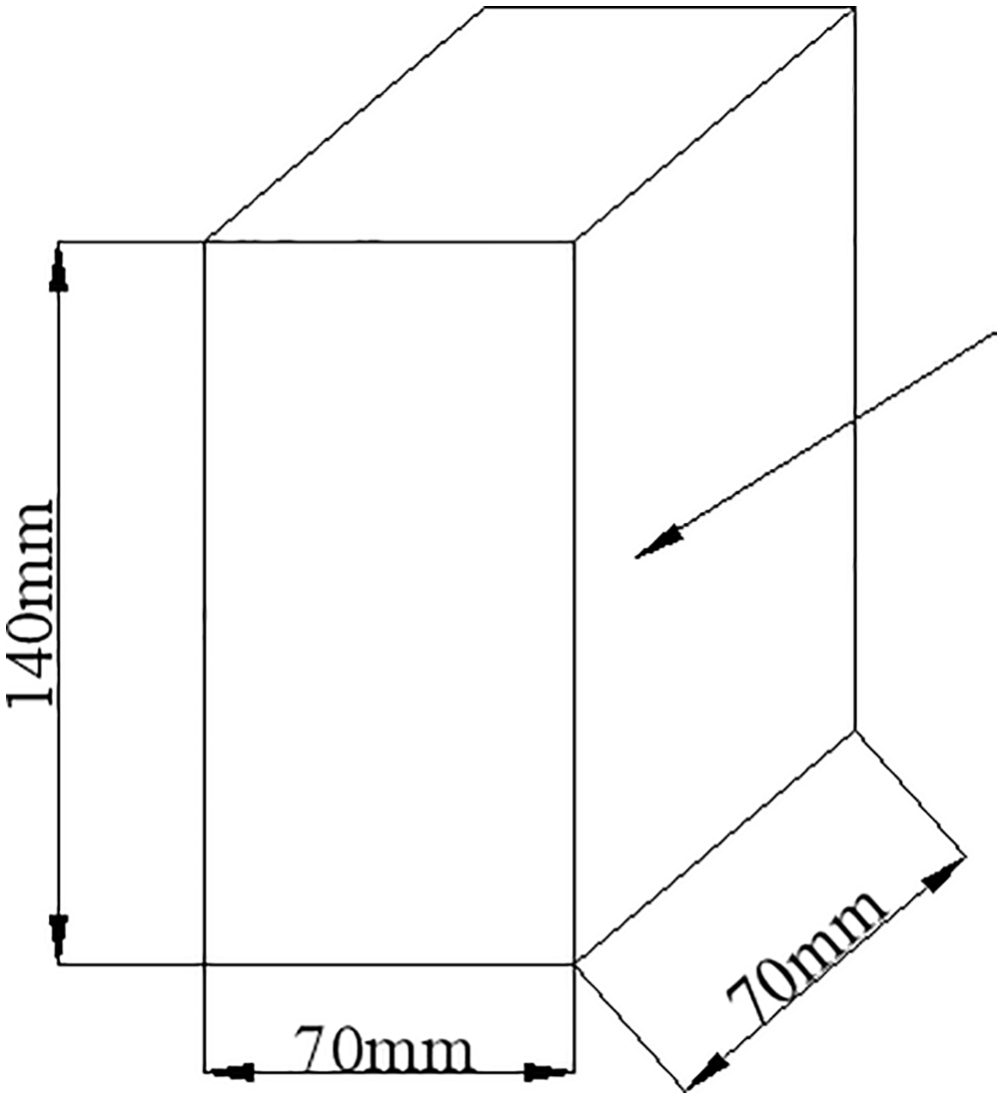
Example of true triaxial tests.

**Fig 11:**
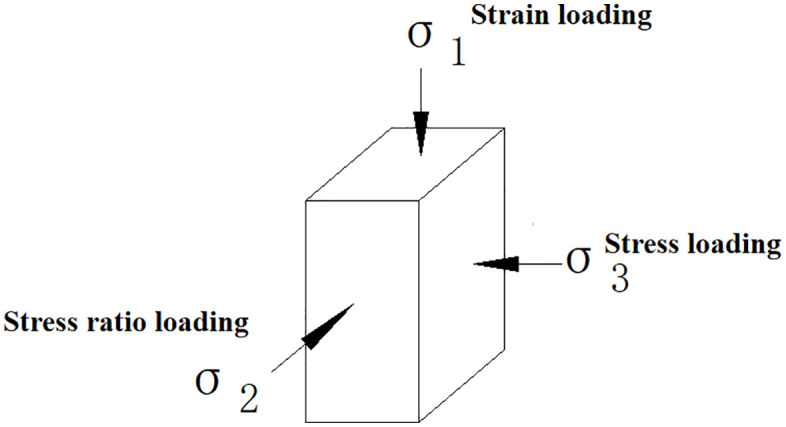
True triaxial test loading method.

**Fig 12:**
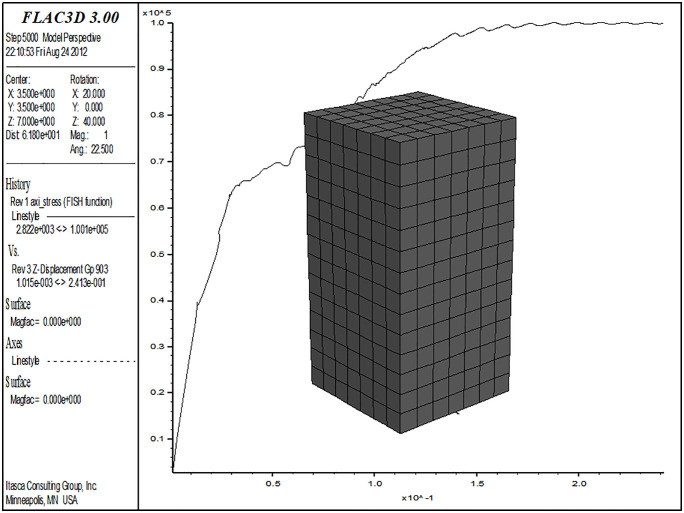
Simulation of true triaxial test in Flac3D.

The sample dimensions are 70 mm x 70 mm x 140 mm. The large principal stress is applied using strain loading, the small principal stress is applied using stress loading, and the medium principal stress is adjusted based on real-time monitoring of the large principal stress. The monitoring and loading procedures are implemented through external functions in FLAC3D. See Figs 10 and 11 for the sample illustrations and loading diagrams, and Fig 12 for the numerical analysis model and the simulated stress-strain relationship curve. During the loading process, the consolidation stress, which is the small principal stress, is $\sigma_{\text{2}}=b\sigma_{\text{1}}+(1-b)\sigma_{\text{3}}$ maintained at a constant level throughout the loading process. The large principal stress is applied at a constant rate, with the compression rate controlled to match that of a true triaxial test, set at 0.05 mm/min. The medium principal stress is loaded by controlling its ratio. During the experiment, the medium principal stress is adjusted in real-time based on the stress changes in the direction of the large principal stress, ensuring that the loading meets specific requirements. The medium principal stress ratios b are set at 0, 0.25,0.5, 0.75, and 1.0. When b = 0, the medium principal stress remains equal to the small principal stress; when b = 1, the medium principal stress remains equal to the large principal stress.

### 3.2. Comparison between the simulation results and experimental results of true triaxial tests on collapsible loess

The data for the examples in this section are derived from the true triaxial tests conducted by the author’s research team [11]. Since the soil samples used in these tests are identical to those in this study, the model parameters used are consistent with those listed in Table 1. The test water content is 15.65%, and the consolidation pressures are 50kPa, 100kPa, 150kPa, 200kPa, and 300kPa.

**Fig 13:**
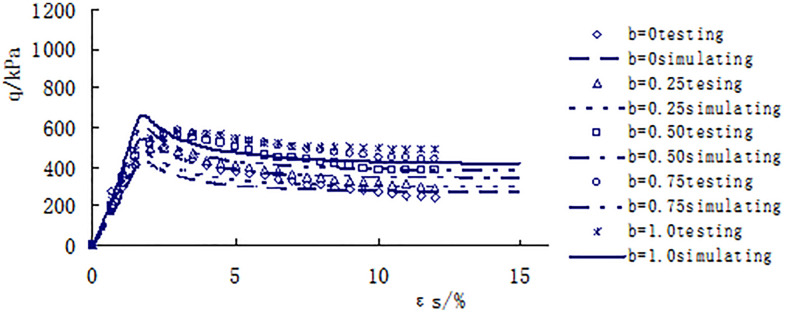
Comparison between measured and calculated values under consolidated confining pressure of 50kPa.

**Fig 14:**
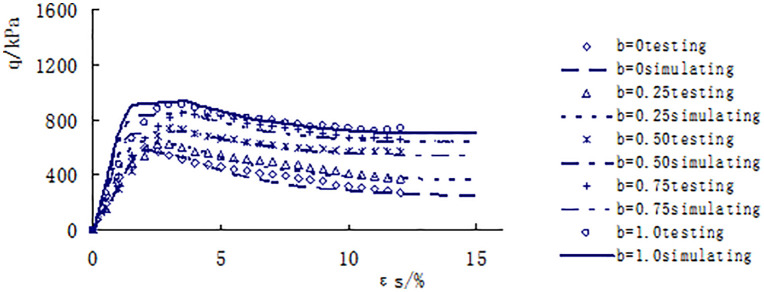
Comparison between measured and calculated values under consolidated confining pressure of 100kPa.

**Fig 15:**
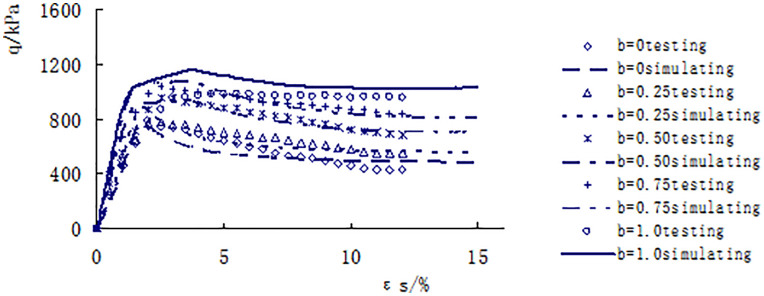
Comparison between measured and calculated values under consolidated confining pressure of 150kPa.

**Fig 16:**
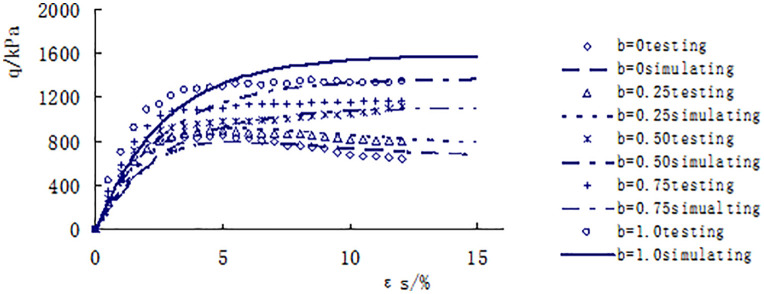
Comparison between measured and calculated values under consolidated confining pressure of 200kPa.

**Fig 17:**
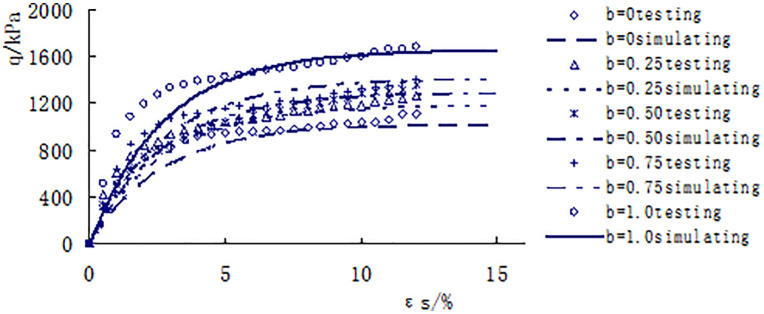
Comparison between measured and calculated values under consolidated confining pressure of 300kPa.

Fig 13–17 shows the comparative analysis curves of numerical simulation and experimental measurement values of different intermediate stress ratios under consolidation pressure of 50kPa, 100kPa, 150kPa, 200kPa and 300kPa.

As shown in Figs 13–17, the strength of loess under different consolidation pressure conditions shows a consistent trend with the increase in the ratio of intermediate principal stress. As this ratio increases, the soil’s strength also increases. Moreover, the numerical simulation results closely match the experimental data, indicating that considering changes in the intermediate principal stress is reasonable and that the proposed strength criterion formula is practical.

A qualitative and semi-quantitative assessment of the agreement between the numerical simulations and experimental data was performed. For all test conditions presented in Figs 13–17, the simulated stress-strain curves closely followed the trends and captured the key features (e.g., peak strength, post-peak behavior, and the effect of the intermediate principal stress ratio b) of the experimental data. To provide a quantitative measure of the fit for the peak strength—a critical parameter in strength criteria, the percentage deviation between the simulated and measured peak deviator stress was calculated for each test. The absolute values of these percentage deviations fell within a range of 3% to 8%, with the majority being below 5%. This demonstrates that the proposed model possesses a high level of accuracy in predicting the strength of structural loess under true triaxial conditions.

## 4. Discussion

### 4.1. Physical Significance and Impact of Model Parameters

The core innovation of the three-dimensional strength criterion proposed in this study (Equation (7) lies in the introduction of the structural state variable *r* Equation (4). This variable is defined as the ratio of the shear strength of structured loess to that of normal consolidated soil under the same spherical stress. This quantitative index directly reflects the “excess strength” provided by the natural structure of loess. As shown in Fig 6 of the original text, the magnitude of the *r* value directly determines the position of the critical state line on the *q*-*p* plane. The higher the *r* value, the higher the strength envelope line.

Furthermore, the establishment of the criteria relies on the three-dimensional extension of the classical strength theory. As shown in Equation (1), by introducing parameters, the slope *M*_c_ of the critical state line of conventional triaxial compression is extended to the general stress state, thereby considering the intermediate principal stress effect. This expansion, combined with the structural nature of the soil, enables the final three-dimensional strength criterion (Equation (11)) to simultaneously reflect the structural strength of the material and the influence of complex stress states.

### 4.2. Comparison with the Classic Mohr-Coulomb Criterion

In engineering practice, the Mohr-Coulomb criterion is still widely applied. However, as shown in the results of this study, the Mohr-Coulomb criterion has obvious limitations: Firstly, it cannot consider the influence of the principal stress on the strength; Secondly, it is difficult to describe the strength characteristics of soil with significant structural properties such as loess, especially under low confining pressure, the Mohr-Coulomb criterion often overestimates or underestimates the strength of structural soil.

This model has made significant progress precisely in these two aspects: Three-dimensional stress consideration: By extending the strength criterion to the three-dimensional stress space through theoretical derivation, the force state of soil in actual engineering can be simulated more realistically. Structural integration: Through the structural variable r, as the structural parameters change, the failure surface shows a dynamic transition from an approximate circle to a curved triangle on the π plane, which theoretically explains the strength change process of structural loess, as shown in Figs 8 and 9 of the original text.

## 5. Conclusion

The structural strength criterion established in this study has been successfully integrated into the wetting-loading constitutive model for loess. Custom subroutines were developed and embedded into the FLAC3D numerical software, validating the rationality and accuracy of the model and demonstrating its feasibility for large-scale numerical simulation.

To account for the influence of intermediate principal stress on soil yield and failure, a three-dimensional strength criterion incorporating structural effects was formulated. This criterion captures the evolution of the failure surface shape under varying structural parameters.

Based on this three-dimensional criterion and the constitutive model, external function programs were developed. The customized model was used to simulate true triaxial tests on loess, and the results showed good agreement with experimental measurements, confirming the validity of the proposed approach.

The structural strength criterion established in this study, which incorporates the effects of intermediate principal stress and soil structure, has been successfully integrated into a constitutive model for loess. The development of custom subroutines enabled its implementation in FLAC3D, and subsequent numerical simulations of true triaxial tests showed excellent agreement with experimental data. This validates the model’s capability to replicate the stress-strain-strength behavior of structural loess under complex three-dimensional stress paths.

Looking forward, this work opens several avenues for further research and practical application.Future studies could focus on extending the model to account for cyclic hydraulic loading and its coupling with more sophisticated hydro-mechanical constitutive frameworks. In terms of engineering practice, the validated model provides a reliable numerical tool for analyzing boundary-value problems critical in loess regions. Establishing standardized protocols for determining the model’s parameters from conventional or in-situ tests will be a key step toward its broader adoption in geotechnical design.
